# Paracetamol clinical dosing routine leads to paracetamol underexposure in an adult severely ill sub-Saharan African hospital population: a drug concentration measurement study

**DOI:** 10.1186/s13104-017-3016-8

**Published:** 2017-12-04

**Authors:** Jeannet C. Bos, Mabor C. Mistício, Ginto Nunguiane, Ron A. A. Mathôt, Reinier M. van Hest, Jan M. Prins

**Affiliations:** 1 Division of Infectious Diseases, Department of Internal Medicine, Academic Medical Centre, University of Amsterdam, Meibergdreef 9, 1105 AZ Amsterdam, The Netherlands; 2 Faculty of Health Sciences, Research Centre for Infectious Diseases (CIDI), Catholic University of Mozambique, Rua Marquês do Soveral 960, C.P. 821, Beira, Mozambique; 3 Division of Clinical Pharmacology, Department of Hospital Pharmacy, Academic Medical Centre, University of Amsterdam, Meibergdreef 9, 1105 AZ Amsterdam, The Netherlands

**Keywords:** Paracetamol, Palliative care, Africa south of the Sahara, Hospital, Oral drug administration, Pharmacokinetics

## Abstract

**Background:**

Hospitals in sub-Saharan Africa (SSA) continue to receive high numbers of severely ill (HIV-infected) patients with physical pain that may suffer from hepatic and renal dysfunction. Paracetamol is widely used for pain relief in this setting but it is unknown whether therapeutic drug concentrations are attained. The aim of this study was to assess the occurrence of therapeutic, sub-therapeutic and toxic paracetamol concentrations in SSA adult hospital population.

**Methods:**

In a cross-sectional study, plasma paracetamol concentrations were measured in patients with an oral prescription in a referral hospital in Mozambique. From August to November 2015, a maximum of four blood samples were drawn on different time points for paracetamol concentration measurement and biochemical analysis. Study endpoints were the percentage of participants with therapeutic (≥ 10 and ≤ 20 mg/L), sub-therapeutic (< 10 mg/L) and toxic (> 75 mg/L) concentrations.

**Results:**

Seventy-six patients with a median age of 37 years, a body mass index of 18.2, a haemoglobin concentration of 10.3 g/dL and an albumin of 29 g/L yielded 225 samples. 13.4% of participants had one or more therapeutic paracetamol concentrations. 86.6% had a sub-therapeutic concentration at all time points and 70.2% had two or more concentrations below the lower limit of quantification. No potentially toxic concentrations were found.

**Conclusions:**

Routine oral dosing practices in a SSA hospital resulted in substantial underexposure to paracetamol. Palliation is likely to be sub-standard and oral palliative drug pharmacokinetics and dispensing procedures in this setting need further investigation.

## Introduction

Health care institutions in sub-Sahara Africa (SSA) continue to receive large numbers of severely ill HIV-infected patients with opportunistic infections and cancer, conditions known to be important causes of physical pain irrespective of treatment with antiretroviral drugs or chemotherapy [1, 2]. In the relative absence of opioid drugs in this region of the world, oral formulations of paracetamol (acetaminophen) are commonly prescribed for the relief of fever and pain. In the so-called WHO Pain Ladder, an analgesic treatment tool for physicians, the use of paracetamol is recommended as a first step [3, 4].

To improve palliative care in SSA health care institutions, a great deal of attention is paid to supply chains of analgesic drugs and training of health care workers on drug prescribing [3]. Information about analgesic drug concentrations is however scarce, whereas this could offer insight on the actual attainment of therapeutic drug concentrations and the appropriateness of existing dosing routines. Since kidney and liver disease are common in the general population as a result of the high prevalence of hypertension and hepatitis B, and over-the-counter drug and prescription medicine misuse is high, such information could also shed light on the occurrence of potentially toxic drug concentrations [5–8].

The aim of this pilot study was to evaluate plasma paracetamol drug concentrations in an adult SSA hospital population during routine oral paracetamol dosing, whilst investigating the occurrence of therapeutic, sub-therapeutic and potentially toxic drug concentrations.

## Methods

### Setting

Mozambique has an estimated adult HIV prevalence of 10.6% [9]. The Beira Central Hospital (HCB) is a 733-bed governmental referral health facility with 260 internal medicine beds, admitting up to 1500 patients monthly. The HIV prevalence on medicine wards is estimated to be at least 75% and up to 30% of patients die during hospital stay [10].

### Study design

The current study was a cross-sectional pilot study and a sub-study of a larger population pharmacokinetic (PPK) study of antibiotics. In this study, pharmacokinetic (PK) data were collected from October 2014 until November 2015 from adult patients admitted to the medicine ward of the HCB, who were being treated with one or more of the following intravenously administered antimicrobials: benzylpenicillin, ampicillin, gentamicin and ceftriaxone.

### Recruitment and data collection

Patients were selected on the basis of use of study antibiotics as documented in a patient’s medication administration record. Inclusion criteria were age ≥ 18 years and being willing and able to give informed consent. Exclusion criteria were the use of drugs known to significantly affect PK of the different study antibiotics, a hemoglobin level ≤ 6 g/dL, and any condition necessitating a blood transfusion, irrespective of hemoglobin level. PPK study participants with any quantitative oral paracetamol prescription were selected for the current sub-study. Only participants with an ‘as needed’ paracetamol prescription (pro re nata: PRN) were excluded.

A participant’s weight and length were measured by the study team and other baseline characteristics and paracetamol dosing information were captured, as documented in a patient’s (medication) record. During 2–3 consecutive days, a maximum of four blood draws were performed for the measurement of antibiotic drug concentrations: a trough level, a peak level and two levels at random time points. The same blood samples were used for the determination of paracetamol concentrations, thus providing random sampling relative to the moment of administration of paracetamol. The dispensing and ingestion of paracetamol were not directly observed. One blood sample was used for the measurement of albumin, liver enzyme and creatinine concentrations. Plasma was stored at − 80 °C in the local research laboratory until shipment on dry ice to the Netherlands for biochemical marker and drug concentration analysis.

Total plasma paracetamol concentrations were measured using a validated colorimetric enzymatic assay with the Cobas Integra 800 System Analyzer (Roche Diagnostics, Mannheim, Germany). The lower limit of quantification was 2 mg/L.

Study endpoints were the percentage of patients with a therapeutic paracetamol concentration defined as > 10 and < 20 mg/L, the percentage of patients with a sub-therapeutic paracetamol concentration defined as < 10 mg/L and the percentage with a drug level that was considered potentially toxic, defined as > 75 mg/L [11–13].

### Data processing and analysis

Data were entered, cleaned and analysed using Excel 2011 (Microsoft, Redmont, WA, USA) and the Excel descriptive statistics add-in tool StatPlus 4.8 (AnalystSoft Inc. Walnut, CA, USA) for quantitative items.

## Results

### Study population

Participants for the current study were selected from the 143 PPK study participants enrolled during the last 4 months of the inclusion period. This resulted in 78/143 (54.6%) patients with an oral paracetamol prescription. Five patients had a PRN prescription and six patients had no blood samples available for drug concentration measurement, leaving the study with a total of 67 participants. A majority of 57/67 participants (85.1%) had a 500 or 1000 mg three times per day prescription (Table 1).

**Table 1:** Characteristics of the study population (n = 67)

Characteristic	
Female sex (n/%)	26 (38.8)
Age (years)	37 (19–77)
Body mass index	18.2 (10.5–25.6)
Haemoglobin (g/dL)	10.3 (6.1–14.4)
Albumin (g/L)	29 (12–44)
GGT (U/L 37C)	65 (7–569)
ALT (U/L 37C)	22 (4–176)
AST (U/L 37C)	41 (14–355)
Creatinine clearance (mL/min)	81 (4–160)
Paracetamol dosing regimen prescribed (n/%)	
3 × 500 mg	47 (70.1)
3 × 1000 mg	10 (14.9)
4 × 500 mg	6 (9.0)
Other	4 (6.0)

Results expressed as median (range) unless specified otherwise. Creatinine clearance estimated using Cockroft-Gault equation

At least one abnormal liver function marker was present in 34/67 (50.7%) participants and 30/67 (44.8%) had an estimated creatinine clearance below 80 mL/min.

### Paracetamol concentrations

**Fig. 1:**
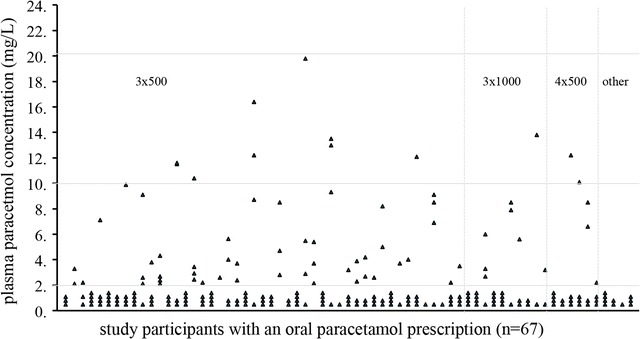
Plasma paracetamol concentrations per participant for different paracetamol-dosing regimens. 3 × 500: 500 mg, three times per day; 3 × 1: 1 g, three times per day; 4 × 500: 500 mg, four times per day

A total of 225 blood samples were available for analysis. Of the 67 participants, 62 (92.5%) had two or more blood samples available for drug concentration measurement (Fig. 1). Plasma paracetamol concentrations were generally low, with 86.6% (58/67) participants having sub-therapeutic levels at all time points and 70.2% (47/67) participants having two or more paracetamol levels below the detection limit. One or more therapeutic drug level were measured in 13.4% (9/67) participants, most of whom had a 500 mg three times/day prescription. The highest concentration measured was 19.8 mg/L and no potentially toxic concentrations were found.

## Discussion

In this pilot study, paracetamol concentrations were measured in a SSA hospital medicine ward population consisting of young, chronically severely ill patients, as illustrated by a low median BMI and a high frequency of anaemia, hypoalbuminaemia, and renal and hepatic dysfunction, as illustrated by the creatinine and liver enzyme concentration results. While employing routine paracetamol dosing practices, paracetamol concentrations were sub-therapeutic, if measurable at all, in the vast majority of patients, even though paracetamol is metabolized by the liver and eliminated by the kidneys. Yet, very few patients had one or more therapeutic paracetamol concentrations. Potentially toxic drug concentrations were not found. One study with healthy volunteers and another with patients under spinal anaesthesia looked into the PK of 1 g of orally administered paracetamol (≈ 15 mg/kg) and found a mean C_max_ of 12.3 and 18.0 mg/L respectively, although with high inter-individual variability [14, 15]. Data from a study in an elderly population with a 1 g three times per day oral regimen rendered a mean trough level of 1.3 mg/L [16]. Based on this evidence, it is conceivable that a substantial part of our study’s drug concentrations would be around or under the lower level of the therapeutic range. Given our random sampling procedures however, the common occurrence of (immeasurably) low paracetamol levels across all samples does not seem to fit the observations in the studies mentioned, even when using a relatively low daily dose of paracetamol. Although the plasma paracetamol concentration is not always predictive of an individual’s pain response, concentrations between 10 and 20 mg/L are often believed to represent the therapeutic range [11, 15]. Our study’s consistent pattern of paracetamol concentrations below 10 mg/L, or even below the level of detection, is therefore worrisome, as it may promote unnecessary pain and fever.

Possible explanations may be found at a patient’s level as well as at the health system level. Interestingly, results from a clinical PK study comparing oral paracetamol absorption in patients while supine and standing demonstrated that in subjects taking a tablet while supine, esophageal transit of tablets was delayed. Paracetamol peak plasma concentrations occurred later while being significantly lower [17]. Our study population largely consisted of weak, bedridden patients who lack consistent assistance with eating, drinking and taking oral medication, and impaired oral drug absorption could therefore have contributed to the low drug concentrations found. In critically ill patients, systemic exposure to paracetamol can also decrease when the volume of distribution increases as a result of fluid shifts and hypoalbuminaemia [18].

Apart from the potential influences of PK processes, shortcomings in the efficiency of the local health system may have played a substantial role in patients not achieving therapeutic paracetamol levels. There is a shortage of motivated, well-trained health care workers, and few nurses may be responsible for large numbers of very ill patients [19]. This can lead to erroneous drug dispensing as well as inconsistent assistance with eating, drinking and taking oral medication.

High in-patient HIV-related morbidity and mortality as well as weaknesses in the health system are not unique to Mozambique, and we suppose that underexposure to oral analgesic drugs is therefore not unlikely to happen in health care institutions across SSA [20].

An important limitation of this study is that drug dispensing and ingestion were not directly observed and that blood samples were drawn at time points relative to the dosing of study antibiotics, and not relative to dosing of paracetamol. Although it was thus not able to relate paracetamol concentrations to actual doses and administration time points, the study’s approach rendered samples that were randomly distributed over the paracetamol-dosing interval, covering peak levels as well as troughs.

## Conclusion

In a chronically and severely ill adult SSA hospital population with a paracetamol prescription, underexposure to paracetamol appears to be common when exercising a routine oral dosing practice. The occurrence of low plasma paracetamol concentrations is likely to lead to sub-optimal palliation. Although this pilot study has limited power to make inferences, the study results do seem to underline a need for a comprehensive oral palliative drug monitoring and evaluation agenda in SSA that addresses analgesic drug therapy at a health system—as well as at a patient level.

## Abbreviations

SSA: sub-Saharan Africa(n)
PPK: population pharmacokinetic(s)
PK: pharmacokinetic(s)
BMI: body mass index
PRN: pro re nata (‘as needed’)
GGT: gamma-glutamyl transferase
AST: aspartate aminotransferase
ALT: alanine aminotransferase
C_max_: peak plasma concentration
